# An assessment of factors associated with quality of randomized controlled trials for smoking cessation

**DOI:** 10.18632/oncotarget.10742

**Published:** 2016-07-20

**Authors:** Hong Fan, Fujian Song, Hai Gu, Jianming Wang, Guizhen Jia, Moyuan Lu, Jiao Qian, Lei Wang, Jiemiao Shen, Zhewen Ren

**Affiliations:** ^1^ Center for Health Policy and Management Research, Nanjing University, Nanjing, P.R.China; ^2^ Department of Social Medicine and Health Education, School of Public Health, Nanjing Medical University, Nanjing, P.R.China; ^3^ Department of Population Health and Primary Care, Norwich Medical School, University of East Anglia, Norwich, UK; ^4^ Department of Epidemiology and Biostatistics, School of Public Health, Nanjing Medical University, Nanjing, P.R.China

**Keywords:** smoking cessation, quality, randomized controlled trials

## Abstract

To reduce smoking-related diseases, a research priority is to develop effective interventions for smoking cessation, and evidence from randomized controlled trials (RCTs) is usually considered to be the most valid. However, findings from RCTs may still be misleading due to methodological flaws. This study aims to assess the quality of 1083 RCTs of smoking cessation interventions in 41 relevant Cochrane Systematic Reviews (CSRs). Logistic regression analysis was performed to identify significant variables associated with the quality of RCTs. It was found that evidence for smoking cessation from RCTs was predominantly from high income countries, and the overall quality was high in only 8.6% of the RCTs. High quality RCTs tended to have a larger sample size, to be more recently published, and conducted in multiple countries belonging to different income categories. In conclusion, the overall quality of RCTs of smoking cessation interventions is far from perfect, and more RCTs in less developed countries are required to generate high grade evidence for global tobacco control. Collaboration between researchers in developed and less developed countries should be encouraged.

## INTRODUCTION

Tobacco use remains the leading preventable cause of premature deaths in the world, and smoking-related illness imposes a heavy economic toll on countries in both direct medical care and lost productivity [1]. While the prevalence of smoking has been declining in developed countries, cigarette smoking remains high, particularly among men, in less developed countries. The World Health Organization estimated that tobacco use is likely to cause over 8 million deaths per year in the next two decades, and more than 80% of these deaths will occur in low and middle income countries (LMICs) [2]. Therefore, one of research priorities is to develop and evaluate smoking cessation interventions, in order to prevent or reduce diseases attributable to tobacco use, in both developed and less developed countries.

Randomized controlled trials (RCTs) can provide valid evidence on the effectiveness of smoking cessation interventions. However, findings from RCTs may be misleading due to methodological flaws, including inappropriate patient allocation, lack of blinding, and imbalanced withdrawals from a study [3]. Risk of bias in RCTs should be carefully assessed, before applying results of RCTs to guide clinical and public health practice [4].

Previous studies found that most research on tobacco control were conducted in high-income countries [5, 6], and the quality of RCTs on non-communicable diseases in less developed countries tended to be lower than those in developed countries [7]. Evidence on the overall quality of RCTs on smoking cessation and associated factors remains scarce. The main purpose of this study is to assess the quality of RCTs of smoking cessation interventions, and to identify associated factors.

## RESULTS

**Figure 1:**
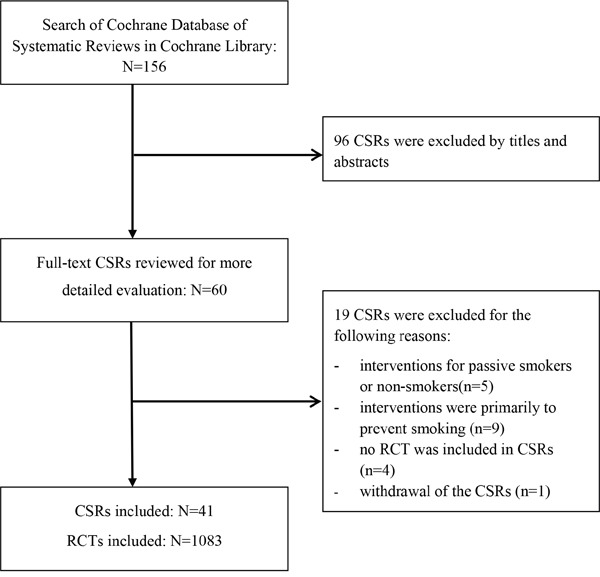
Selection of relevant Cochrane Systematic Reviews (CSRs)

The process of the selection of relevant Cochrane Systematic reviews (CSRs) is shown in Figure 1. The initial search identified 156 CSRs from a total of 9301 records in the Cochrane Database of Systematic Reviews. We excluded 96 CSRs after screening their titles and abstracts, and excluded 19 CSRs after checking full text details. A total of 41 CSRs met the inclusion criteria and made up the dataset [8–48].

The main characteristics of the included CSRs are summarized in Table 1. Corresponding authors of the included CSRs were all from institutions in high-income countries. Smoking cessation interventions evaluated were behavioral therapy in 13 (31.7%), pharmaceutical aids in 6 (14.6%), psychosocial interventions in 5 (12.2%), tobacco control policies in 2 (4.9%), nicotine vaccines in 1 (2.4%), self-help in 1 (2.4%), and mixed interventions in 13 (31.7%). Of the 41 CSRs, 28 (68.3%) were updated after 2012. The 41 CSRs included a total of 1083 RCTs. The median number of RCTs included in these CSRs was 21 (interquartile range 7 to 35). Language restriction was explicitly applied in only three CSRs (including RCTs published in English or Chinese).

**Table 1:** The characteristics of the included cochrane systematic reviews (CSRs)

	Number of CSRs (%)
**Total**	41
**Country of CSR corresponding authors**	
High-income countries	41 (100.0%)
**Type of smoking cessation interventions**	
Policies	2 (4.9%)
Behavioural therapy	13 (31.7%)
Pharmaceutical quit aids	6 (14.6%)
Nicotine vaccines	1 (2.4%)
Psychosocial interventions	5 (12.2%)
Self-help	1 (2.4%)
Mixed	13 (31.7%)
**Year of updated to:**	
2010	2 (4.9%)
2011	2 (4.9%)
2012	9 (22.0%)
2013	14 (34.1%)
2014	8 (19.5%)
2015	6 (14.6%)
**Number of RCTs included**:	
1 – 9	13 (31.7%)
10 – 19	6 (14.6%)
20 – 29	9 (22.0%)
30 – 39	4 (9.8%)
≥40	9 (22.0%)
**Any language restriction:**	
No	37 (90.2%)
Yes	3 (7.3%)
Unclear	1 (2.4%)

### The main characteristics of RCTs and risk of specific biases

Of the 1083 RCTs included in the 41 CSRs, 96.1% were conducted in high-income countries, 2.8% in LMICs, and 1.1% in multiple-income countries (in both high-income and LMICs) (Table 2). Most of the included RCTs were published in English (96.9%), and only 3.1% in other languages (Chinese, Japanese, French, Germany, etc.). For the 10 RCTs conducted in China, 6 were published in Chinese language. Sample sizes of the RCTs ranged from 9 to 42277 (median 280, interquartile range: 120 to 719), and the sample size was ≥700 in 25% of the RCTs. The number of RCTs included in the CSRs was increasing over time, and more than half (62.3%) were published since 2000. Unpublished data were obtained for 92 of the included RCTs (8.5%).

**Table 2:** The number and proportion of randomized controlled trials (RCTs) with low risk of bias by study characteristics

	Sequence generation	Allocation concealment	Blinding	Incomplete outcome	Reporting
**Total (n=1083)**	441(40.7%)	331(30.6%)	253(23.4%)	599(55.3%)	115(10.6%)
**Country income levels**					
High-income (n=1041)	419(40.2%)	312 (30.0%)	242(23.2%)	574(55.1%)	108(10.4%)
LMICs (n=30)	12 (40.0%)	8 (26.7%)	7 (23.3%)	17 (56.7%)	3 (10.0%)
Multiple-income (n=12)	10 (83.3%)	11 (91.7%)	4 (33.3%)	8 (66.7%)	4 (33.3%)
**Publication language of RCTs**					
English (n=1049)	437(41.7%)	328 (31.3%)	245(23.4%)	589(56.1%)	114(10.9%)
Other languages (n=34)	4 (11.8%)	3(8.8%)	8 (23.5%)	10(29.4%)	1 (2.9%)
**Total sample size of RCTs**					
< 100 (n=215)	61 (28.4%)	38 (17.7%)	35 (16.3%)	102(47.4%)	37 (17.2%)
100 –299 (n=352)	155(44.0%)	108 (30.7%)	91 (25.9%)	192 (54.6%)	40 (11.4%)
300 – 699 (n=242)	108 (44.6%)	83 (34.3%)	64 (26.5%)	133 (55.0%)	18 (7.4%)
≥ 700 (n=274)	117 (42.7%)	102 (37.2%)	63 (23.0%)	172 (62.8%)	20 (7.3%)
**Year of publication of RCTs**					
Before 1990 (n=135)	13 (9.6%)	12 (8.9%)	36 (26.7%)	60 (44.4%)	19 (14.1%)
1990-1999 (n=273)	66 (24.2%)	56 (20.5%)	55 (20.1%)	131 (48.0%)	14 (5.1%)
2000-2009 (n=484)	245 (50.6%)	185 (38.2%)	114(23.6%)	294 (60.7%)	51 (10.5%)
Since 2010 (n=191)	117 (61.3%)	78 (40.8%)	48 (25.1%)	114 (59.7%)	31 (16.2%)
**Publication status**					
Published(n=991)	390 (39.4%)	292 (29.5%)	234(23.6)%	565 (57.0%)	103(10.4%)
Unpublished(n=11)	3 (27.3%)	3 (27.3%)	3 (27.3%)	4 (36.4%)	0 (0.0%)
Published and unpublished(n=81)	48 (59.3%)	36 (44.4%)	16 (19.8%)	30 (37.0%)	12 (14.8%)

**Note:** RCTs conducted in “multiple-income” refers to RCTs that recruited participants in both high-income countries and LMICs.

The proportion of RCTs with a low risk of bias was 40.7% in terms of sequence generation, 30.6% in terms of allocation concealment, 23.4% in terms of blinding, 55.3% in terms of incomplete outcome, and 10.6% regarding reporting bias (Table 2). The quality regarding to sequence generation, allocation concealment and incomplete outcome was similar for RCTs conducted in high-income countries and in LIMCs, although it was highest when studies were conducted in mixed-income countries (that is, in both developed and less developed countries). The quality tended to be higher for RCTs published in English, compared with those published in other languages. With certain exceptions, the quality of RCTs tended to be positively associated with larger sample sizes and more recent publications. The use of both published and unpublished data was associated with low risk of bias in terms of sequence generation and allocation concealment, although the association was not consistent for other quality domains (see Table 2).

### Overall quality of RCTs and related factors

Defined as at least 4 of the 5 quality domains being low risk of bias, the overall quality was high in only 93 (8.6%) of the 1083 RCTs (Table 3). The proportion of high quality RCTs was 33.3% in RCTs conducted in multiple income countries, compared with 8.3% in RCTs from high-income countries and 10.0% in LMICs (P=0.008). There were no significant differences in the overall quality regarding the publication language (P=0.109) and data publication status (P=0.699) (Table 3). However, there was a clear association between the overall quality of RCTs and sample size (P=0.010). In addition, the proportion of high quality RCTs was noticeably increasing over time, from 1.5% in RCTs published before 1990, to 4.0% between 1990 and 1999, 10.7% between 2000 and 2009, and 14.7% since 2010 (P<0.001).

**Table 3:** The characteristics of randomised controlled trials (RCTs) included in the cochrane systematic reviews (CSRs) by quality

	Overall quality of RCTs		*χ*^2^	*P value*
	Low	High		
**All included RCTs (1083)**	990 (91.4%)	93 (8.6%)		
**Country income levels**				
High-income (n=1041)	955 (91.7%)	86 (8.3%)	9.578	0.008
LMICs (n=30)	27 (90.0%)	3 (10.0%)		
Multiple-income (n=12)	8 (66.7%)	4 (33.3%)		
**Published in English**				
Yes (n=1049)	956 (91.1%)	93 (8.9%)	3.297	0.109
No (n=34)	34 (100.0%)	0 (0.0%)		
**Total sample size**				
< 100 (n=215)	208 (96.7%)	7 (3.3%)	11.319	0.010
100 –299 (n=352)	322 (91.5%)	30 (8.5%)		
300 – 699 (n=242)	217 (89.7%)	25 (10.3%)		
≥ 700 (n=274)	243 (88.7%)	31 (11.3%)		
**Year of publication**				
Before 1990 (n=135)	133 (98.5%)	2 (1.5%)	27.748	<0.001
1990-1999 (n=273)	262 (96.0%)	11 (4.0%)		
2000-2009 (n=484)	432 (89.3%)	52 (10.7%)		
Since 2010 (n=191)	163 (85.3%)	28 (14.7%)		
**Publication status**				
Published(n=991)	908 (91.6%)	83 (8.4%)	0.718	0.699
Unpublished(n=11)	10 (90.9%)	1 (9.1%)		
Published and unpublished(n=81)	72 (88.9%)	9 (11.1%)		

**Note:** High quality research is defined as at least 4 of the 5 quality items being low risk of bias.

We performed multivariable logistic regression analysis to explore factors associated with the overall quality of RCTs (Table 4). The dependent variable was the high overall quality of RCTs, defined as at least four of the five bias items being low. The overall quality of RCTs was higher (that is, at low risk of bias) in RCTs with larger sample sizes (P=0.031), published more recently (P<0.001) and conducted in multiple countries belonging to different income categories (P=0.020).

**Table 4:** Association between overall quality of RCTs and selected study characteristics: results of multi-variable logistic regression analysis

Variables	Odds ratio (95% CI)	P-value
**Country income levels:** Multiple-income (1) vs. high-income or LMICs (0)	4.374 (1.259, 15.202)	0.020
**Sample size:** Sample size ≥300 (1) vs. <300 (0)	1.625 (1.046, 2.523)	0.031
**Year of publication:** Published since 2000 (1) vs. before 2000 (0)	3.888 (2.130, 7.099)	<0.001

**Note:** Dependent variable is defined as at least 4 of the 5 quality items being low risk of bias (0 for high risk, 1 for low risk).

## DISCUSSION

Effects of smoking cessation treatments are usually small and relapse is common [49]. To improve the smoking cessation success, smokers who want to quit should be treated with the most effective smoking cessation interventions [50]. The CSRs included in the current study evaluated a range of smoking cessation interventions, including behavioral, pharmacological, psychosocial, tobacco control policy, and mixed interventions.

RCTs may provide high-quality evidence, but they can also be graded down because of flaws in design, conduct and reporting. The current study found that less than 50% of the included RCTs had low risk of bias in specific quality domains, except for the bias due to incomplete outcome data (55.3%). It was noted that the proportion of RCTs with unclear or high risk of reporting bias was as high as 89.4%, indicating the existence of publication bias due to the tendency that significant results were more likely to be published [51–53].

In accordance with findings from previous studies [54, 55], the quality of the included RCTs has been improving over time, and was positively associated with a larger sample size. In addition, the quality of RCTs conducted in multiple nations belonging to different income groups was higher than those conducted in either high-income countries or in LMICs. However, the current study found no significant difference in quality between RCTs in LMICs and those in high-income countries, in contrast to findings from a previous study [7]. It should be acknowledged that the number of RCTs from LMICs in this study is very small (n=30), and reported quality may not necessarily reflect the true quality of trials [53].

This study found that most of the include RCTs were from high-income countries (96.1%), and were published in English (96.9%). The possible explanations include lack of research in LMICs, and researchers with English writing capability may have a strong presence in research activities for smoking cessation intervention. Even when studies had been conducted in LMICs and/or by non-English speaking researchers, they might not be published, or published in journals that are not indexed in the widely used bibliographic databases such as MEDLINE and EMBASE. It is unclear whether and about the extent to which RCTs conducted in LMICs or published in languages other than English might have been missed from CSRs. Although the number of RCTs from LMICs (n=30) or published in non-English languages (n=34) was very small in the current study, we found no significant difference in quality between RCTs conducted in developed and less developed countries, and between RCTs published in English and those published in other languages.

Clearly, current smoking cessation and tobacco control practice in less developed LMICs will have to be based mainly on research from high-income developed countries. This raises a question about whether research evidence from high-income countries could be generalizable to LMICs. Generalizability or external validity of findings from RCTs is often context dependent, and questionable across different settings or countries [56, 57]. For example, studies in the United States on average reported smaller effects of cardiorenal drugs compared with those conducted in other countries [58]. A study found that RCTs from less developed countries tended to report more favorable results than those from developed countries [53]. Because of doubtful generalizability of research from developed countries, evidence from local research may be more acceptable by health professionals in LMICs [59]. Scarce in evidence from RCTs will affect the local adoption and implementation of the interventions for smoking cessation in LMICs [60]. However, available evidence regarding the generalizability of research from developed to less developed countries is still very limited, and further research in this area is required.

Given the clear lack of relevant research in LMICs, it is important that more RCTs with high quality are conducted in LMICs to provide research evidence on smoking cessation interventions, which will contribute to improve representativeness and generalizability of high quality evidence from resources poor settings, and to encourage the researchers’ endeavor in tackling tobacco epidemic in LMICs. For researchers in LMICs, poor access to research funding and challenges with the publication of research may hinder their research activities. The development of research capacity in less developed countries will contribute to the control of diseases globally. Although the flow of research evidence is currently mainly from developed to less developed countries, it has been recently emphasized that high quality research in LMICs may also benefit developed countries [61]. According to findings from the current study and a previous study [7], collaboration between researchers in developed and less developed countries will be more likely to generate higher quality evidence. International and national research funding bodies need to encourage and support more studies of smoking cessation and tobacco control that are collaboratively conducted by researchers in developed and less developed countries.

### Limitations

We only assessed RCTs included in Cochrane systematic reviews, and some relevant RCTs of smoking cessation interventions might have been missed. For example, the CSRs may have excluded some low quality RCTs from LMICs and non-English speaking countries that mislabeled study designs or did not adhere to the CONSORT guidelines for reporting [62]. If this is the case, our analysis may have overestimated the quality of RCTs on smoking cessation. The assessment of quality and risk of bias was based on what is reported by authors, and the actual conduct may be different. In addition, we did not analysis the primary outcome on smoking cessation interventions, which will be the focus of a further study.

## CONCLUSION

The evidence from RCTs was predominantly from high income countries and published in English language. The overall quality of RCTs of smoking cessation interventions is far from perfect, and the quality of RCTs was positively associated with a larger sample size and more recent years of publication. More RCTs with low risk of bias are required in LMICs to generate high grade evidence for global tobacco control. Collaboration between researchers in developed and less developed countries should be encouraged.

## MATERIALS AND METHODS

### Search methods for identifying studies

Cochrane Database of Systematic Reviews in Cochrane Library (Issue 3 of 12, 2016) was searched to identify eligible CSRs. The search strategy used the following combination terms: 'smok* OR tobacco OR cigarette* OR nicotine' in Title, Abstract, or Keywords. Identified CSRs were transferred into an Endnote database.

### Selection of studies

Figure 1 shows the process of selection of CSRs. We first examined titles and abstracts of CSRs, and then checked full text details for those that were possibly eligible. Eligible CSRs were those updated since 2010 and evaluated smoking cessation interventions for current smokers. We exclude CSRs that focused on interventions exclusively for passive smoking, and interventions for the primary prevention of tobacco use. There was no restriction on the length of follow up and the primary outcome measures. Two researchers (HF and JW) applied the inclusion and exclusion criteria to select relevant CSRs, and a third reviewer (FS) was involved when it was difficult to decide the eligibility of a CSR.

### Data extraction and management

Data extraction was conducted by two researchers (JS and LW) and then checked by a third researcher (HF). Any disagreement was resolved by discussion. We extracted data on the following items from the included CSRs:

- Year as up-to-date
- Country of the corresponding author of CSRs
- Language restrictions for study inclusion
- Type of smoking cessation interventions

From RCTs included in the CSRs, we extracted data on the following elements:

- Year of publication
- Sample size
- Country origin
- Publication language
- Publication status
- Results of risk of bias assessment

### Assessment of risk of bias in included RCTs

Risk of bias in all RCTs included in CSRs was assessed in terms of six quality domains: (1) the adequacy of sequence generation, (2) allocation concealment, (3) blinding of patients, care providers and outcome assessors, (4) incomplete outcome data, (5) free of selective outcome reporting, and (6) free of other bias, using the Cochrane Collaboration's tool for assessing risk of bias [4]. For each of the six domains, risk of bias could be judged as being high, low, or unclear. Because risk of other biases (the last item) was inconsistently assessed in CSRs, we used results of risk of bias assessment for the first five domains. In this study, the quality of a RCT was considered to be high, if risk of bias was low for at least 4 of the 5 quality domains.

### Data synthesis and analysis

We summarized the extracted data from the included CSRs and RCTs by tabulations. RCTs included in the relevant CSRs were grouped by trial characteristics. According to the gross national income (GNI) per capita in 2014, the World Bank currently classified countries as high-income (≥$12,736), middle-income (from $1,046 to $12,735), and low-income (≤$1,045) [63]. Because of the small number of RCTs from low-income and middle-income countries, we combined low-income and middle-income countries as low-and-middle-income countries (LMICs) in analysis.

Normality of distribution was determined using QQ plots and Kolmogorov–Smirnov test. For the comparison of categorical variables, Pearson's chi-square test or Fisher's exact test was used as appropriate. To explore the factors associated with risk of bias of included RCTs, we conducted multi-variable logistic regression analyses. Two-tailed P values of less than 0.05 were considered statistically significant. In the logistic regression model, we adopted the Enter Method to achieve a final model. The standard for the variable inclusion was based on SLE=0.05, and the exclusion standard was SLS=0.10. All data were processed using the program SPSS 18.0 (SPSS, Inc., Chicago, IL, USA) and Epidata 3.1.
